# Transcriptomic profiling suggests candidate molecular responses to waterlogging in cassava

**DOI:** 10.1371/journal.pone.0261086

**Published:** 2022-01-21

**Authors:** Min Cao, Linling Zheng, Junyi Li, Yiming Mao, Rui Zhang, Xiaolei Niu, Mengting Geng, Xiaofei Zhang, Wei Huang, Kai Luo, Yinhua Chen

**Affiliations:** 1 Key Laboratory of Sustainable Utilization of Tropical Biological Resources of Hainan Province, Haikou, China; 2 School of Tropical Crops, Hainan University, Haikou, China; 3 School of Life Sciences, Hainan University, Haikou, China; 4 Alliance of Bioversity International and the International Center for Tropical Agriculture (CIAT), Cali, Colombia; 5 Hainan University Archives, Haikou, the People’s Republic of China; University of Agricultural Sciences, INDIA

## Abstract

Owing to climate change impacts, waterlogging is a serious abiotic stress that affects crops, resulting in stunted growth and loss of productivity. Cassava (*Manihot esculenta* Grantz) is usually grown in areas that experience high amounts of rainfall; however, little research has been done on the waterlogging tolerance mechanism of this species. Therefore, we investigated the physiological responses of cassava plants to waterlogging stress and analyzed global gene transcription responses in the leaves and roots of waterlogged cassava plants. The results showed that waterlogging stress significantly decreased the leaf chlorophyll content, caused premature senescence, and increased the activities of superoxide dismutase (SOD), catalase (CAT) and peroxidase (POD) in the leaves and roots. In total, 2538 differentially expressed genes (DEGs) were detected in the leaves and 13364 in the roots, with 1523 genes shared between the two tissues. Comparative analysis revealed that the DEGs were related mainly to photosynthesis, amino metabolism, RNA transport and degradation. We also summarized the functions of the pathways that respond to waterlogging and are involved in photosynthesis, glycolysis and galactose metabolism. Additionally, many transcription factors (TFs), such as MYBs, AP2/ERFs, WRKYs and NACs, were identified, suggesting that they potentially function in the waterlogging response in cassava. The expression of 12 randomly selected genes evaluated via both quantitative real-time PCR (qRT-PCR) and RNA sequencing (RNA-seq) was highly correlated (R^2^ = 0.9077), validating the reliability of the RNA-seq results. The potential waterlogging stress-related transcripts identified in this study are representatives of candidate genes and molecular resources for further understanding the molecular mechanisms underlying the waterlogging response in cassava.

## 1. Introduction

Waterlogging, or soil flooding, is estimated to affect more than 17 million km^2^ of land area per year worldwide. Waterlogging events are expected to increase in frequency, severity, and unpredictability in the future because of global climate change [1]. It has been reported that the intensification of rainfall and evaporation in response to global warming will cause wet regions such as most tropical and subtropical zones to experience waterlogging [2]. Approximately 10% of irrigated farmlands suffer from frequent waterlogging, resulting in substantial yield losses (from 40 to 80%) [3, 4]. In China, losses in crop production due to flooding were second to those due to drought in 2013, accounting for more than RMB 300 billion Yuan [5]. This abiotic stress also causes problems for agricultural production in Australia, North America and Central Europe, especially in regions with heavy-textured soils [6].

The gas diffusion rate in water is much lower than that in air, which is a major determinant of the adverse effects of waterlogging. This low diffusion rate leads to reduced concentrations of oxygen in the root zone, limiting mitochondrial aerobic respiration, supplying energy for nutrient uptake and transport, and causing energy loss [7]. Waterlogging stress further decreases plant shoot metabolism, stomatal conductance, hydraulic conductance, transpiration, respiration and photosynthesis, which manifest as stunted growth and reduced biomass accumulation [8]. Obtaining sufficient knowledge about the mechanisms that drive waterlogging tolerance in plants to develop stress-tolerant crops and anticipate ecosystem changes is an enormous challenge for the plant science research community.

To cope with anaerobiosis due to waterlogging and to regulate different adaptive responses, plants modulate various transcriptional and metabolic changes [9–12]. A major plant response to soil waterlogging is the metabolic switch from aerobic respiration to anaerobic fermentation [13]. This switch involves metabolic adaptations such as induced expression of fermentation pathway enzymes, which leads to a rapid reduction in cellular adenosine triphosphate (ATP) levels. Plants have also developed a series of antioxidant mechanisms to defend themselves against oxidative stress. Antioxidant enzymes such as superoxide dismutase (SOD), catalase (CAT), ascorbate peroxidase (APX), and glutathione reductase (GR) have been reported to remove toxic oxygen substances and prevent or reduce cell damage in many plant species [14]. These reports strongly suggest that the regulation of the waterlogging response in plants involves more than a simple adaptation and is far more complex than anticipated for many years.

Cassava (*Manihot esculenta* Grantz) is considered an important cash and biofuel crop species in Asia, Latin America and Africa because of its starchy roots, making it critical for food security and economic development [15]. This species has been reported to be very drought tolerant; moreover, it can also use light and water resources efficiently and is tolerant to heat [16]. However, existing cassava cultivars that are injured by waterlogging can sometimes never fully recover, and there have been few studies on the adaptations of cassava to waterlogging stress. Globally, although cassava is grown across a wide range of environments, the majority of this species is cultivated in areas where the rainfall is more than 700 mm per year [17]; therefore, cassava is subjected to excess rainfall during the summer rainy season. RNA sequencing (RNA-Seq) has been used as an efficient approach to understanding transcriptome profiles [18]. To better understand the molecular mechanisms underlying the response of cassava to soil waterlogging, the transcriptional profiles of waterlogged cassava roots were analyzed via RNA-seq. Genes expressed in the control and treatment groups were compared to determine the species-specific responses of cassava and to identify genes or strategies associated with waterlogging resistance.

## 2. Materials and methods

### 2.1 Plant growth and waterlogging treatment

The cassava cultivar South China 6068 (SC6068), which is a common cultivar, was used in this study. Cassava plants were propagated clonally from cuttings of parental stems that had at least two nodes and were approximately 8 cm in length. The plants were grown in plastic pots (12 cm in height, 15 cm in diameter) containing a 2:1 mixture (V/V) of potting soil and vermiculite. One plant was grown in each pot. The plants were grown in a glasshouse under natural lighting and temperature (average daily temperature of 28–30°C) at Hainan University (20° 50’ N and 108° 38’ E). At 45 days after the cuttings were planted, those at the same developmental stage were waterlogged. Briefly, the plants were transplanted into plastic containers (62 cm × 36 cm × 42 cm) that were filled with water until the water level was approximately 3–4 cm above the soil surface. Waterlogging treatments were maintained for 6 days. The plants were separated into two groups. Three plants were randomly selected for each group, the leaves and roots of each plant were harvested for RNA extraction, and the remaining samples were used for physiological measurements. Four different types of samples were taken: leaves under waterlogged conditions (WL), roots under waterlogged conditions (WR), leaves under nonwaterlogged conditions (CL) and roots under nonwaterlogged conditions (CR). All the samples were immediately frozen in liquid nitrogen and stored at -80°C until use. All of the waterlogging treatments were performed with three independent biological replicates.

### 2.2 Measurements of chlorophyll content and maximum quantum efficiency of photosystem II (PSII) (*Fv/Fm*)

To estimate the relative chlorophyll content in the leaves of waterlogged plants, the chlorophyll contents were measured using a SPAD-502 Plus portable chlorophyll meter (Konica Minolta, Japan), which calculates a relative chlorophyll content value (SPAD) from the ratio of optical absorbance at 650 nm to that at 940 nm; major veins and areas of obvious visual damage were avoided. An average relative chlorophyll content was obtained for each leaf sample. The *Fv/Fm* values were measured using a handheld fluorometer (FluorPen FP100, Photon Systems Instruments, Czech Republic).

### 2.3 Measurements of enzyme activity

Colorimetric assays were used to measure the contents of peroxidase (POD), CAT and SOD (the kit was purchased from Beijing Solarbio Science & Technology Co., Ltd., Beijing, China). A sample of leaves (0.1 g) without the midrib was thoroughly ground with a cold mortar and pestle in an ice bath. The grinding medium consisted of 1 mL of phosphate buffer plus homogenizing glass beads. The homogenate was centrifuged for 10 min at 8500 rpm and 4°C. The supernatant in turn constituted the crude enzyme extract and was used to determine enzyme activity. The absorbance of the reaction mixture was determined by using an Infinite M200 PRO instrument (Tecan, Switzerland).

### 2.4 Procedures of RNA-sequencing

Library construction, quality detection and Illumina sequencing were carried out by Beijing Biomarker Cloud Technology Co., Beijing, China (www.bmkcloud.com). In total, 1 μg of RNA per sample was used as input material for the RNA sample preparations. Sequencing libraries were generated using a NEBNext Ultra^TM^ RNA Library Prep Kit for Illumina (NEB, USA) according to the manufacturer’s recommendations, and index codes were added to attribute sequences to each sample. The adaptor sequences and low-quality sequence reads were removed from the data sets. Raw sequences were transformed into clean reads after data processing. Clean data (clean reads) were obtained from the raw data by removing reads containing adapters, reads containing poly-N sequences and low-quality reads and then mapped to the reference genome sequence. Only reads with a perfect match or one mismatch were further analyzed and annotated based on the reference genome. Hisat2 software was used to map the reads to the reference (https://www.ncbi.nlm.nih.gov/assembly/GCA_013618965.1). The accession numbers of the transcriptome data was PRJNA699429, which were deposited in the NCBI Sequence Read Archive (SRA) database.

### 2.5 Gene annotation and pathway analysis

For annotations, all unigenes, which were proven to be longer than 200 bp, were subjected to a BLAST search (E-value < 1e^-5^) against the NCBI nonredundant (NR) protein database [19], manually annotated and reviewed protein sequence database Swiss-Prot [20], Gene Ontology (GO) [21], Clusters of Orthologous Groups of proteins (KOG/COG) [22, 23], Protein family (Pfam) and Kyoto Encyclopedia of Genes and Genomes (KEGG) databases [24, 25].

### 2.6 Differential expression analysis

Gene expression levels in each sample was analyzed by using FPKM (fragments per kilobase of exons model per million mapped reads) method [26]. Differential expression analyses of genes between two groups of comparison were performed using the DESeq2 package for FPKM data with biological replicates [27]. The *p* values were corrected using the Benjamini and Hochberg’s method to control the false discovery rate (FDR). Genes differentially expressed with at least 2-fold change (i.e., the absolute value of log_2_ Fold change ≥ 1.0) and a FDR corrected *p*-value of < 0.01 found by DESeq were considered as differentially expressed genes (DEGs). KOBAS software was used to test the statistical enrichment of differentially expressed genes in KEGG pathways [28]. The KEGG pathway with a FDR corrected *p*-value of < 0.05 were considered as enrichment in DEGs.

### 2.7 Quantitative real-time PCR (qRT-PCR) analysis

The expression patterns of twelve genes were analyzed via qRT-PCR. A pair of primers for each gene was designed on the basis of content from the NCBI database (https://www.ncbi.nlm.nih.gov/). The primer pairs are listed in S1 Table. Approximately 1 μg of isolated total RNA was used to generate cDNA using a reverse transcriptase kit (Thermo, USA). qRT-PCR was then performed using a 7500 Real Time PCR System with a total reaction volume of 20 μL, which consisted of 2 μL of cDNA template, 0.4 μL of forward and reverse primers (10 μM each), 10 μL of qPCR Master Mix, 0.4 μL of Rox and 6.8 μL of sterilized ddH_2_O. The PCR conditions were as follows: 95°C for 30 s; 40 cycles of 95°C for 5 s, 60°C for 34 s, and 95°C for 15 s; and 60°C for 1 min. The expression abundances of 12 genes were determined based on the ΔΔCT method described by Schmittgen and Livak [29], and relative changes in gene expression from the qRT-PCR experiments were calculated using *elongation factor 1 alpha* (*EF1α*) as a reference gene. *EF1α* was validated as one of two most stably expressed genes across different tissues and developmental stages in cassava [30]. Three biological replicates and three technical replicates of each group were assessed.

### 2.8 Statistical analysis

All of the experiments used for data comparisons were repeated three times. The statistics were analyzed via analysis of variance (ANOVA) followed by Duncan’s new multiple range test with SPSS version 20.0. The significance level was set to *P* < 0.05.

## 3. Results

### 3.1 Morphological and physiological changes in cassava in response to waterlogging stress

**Fig 1:**
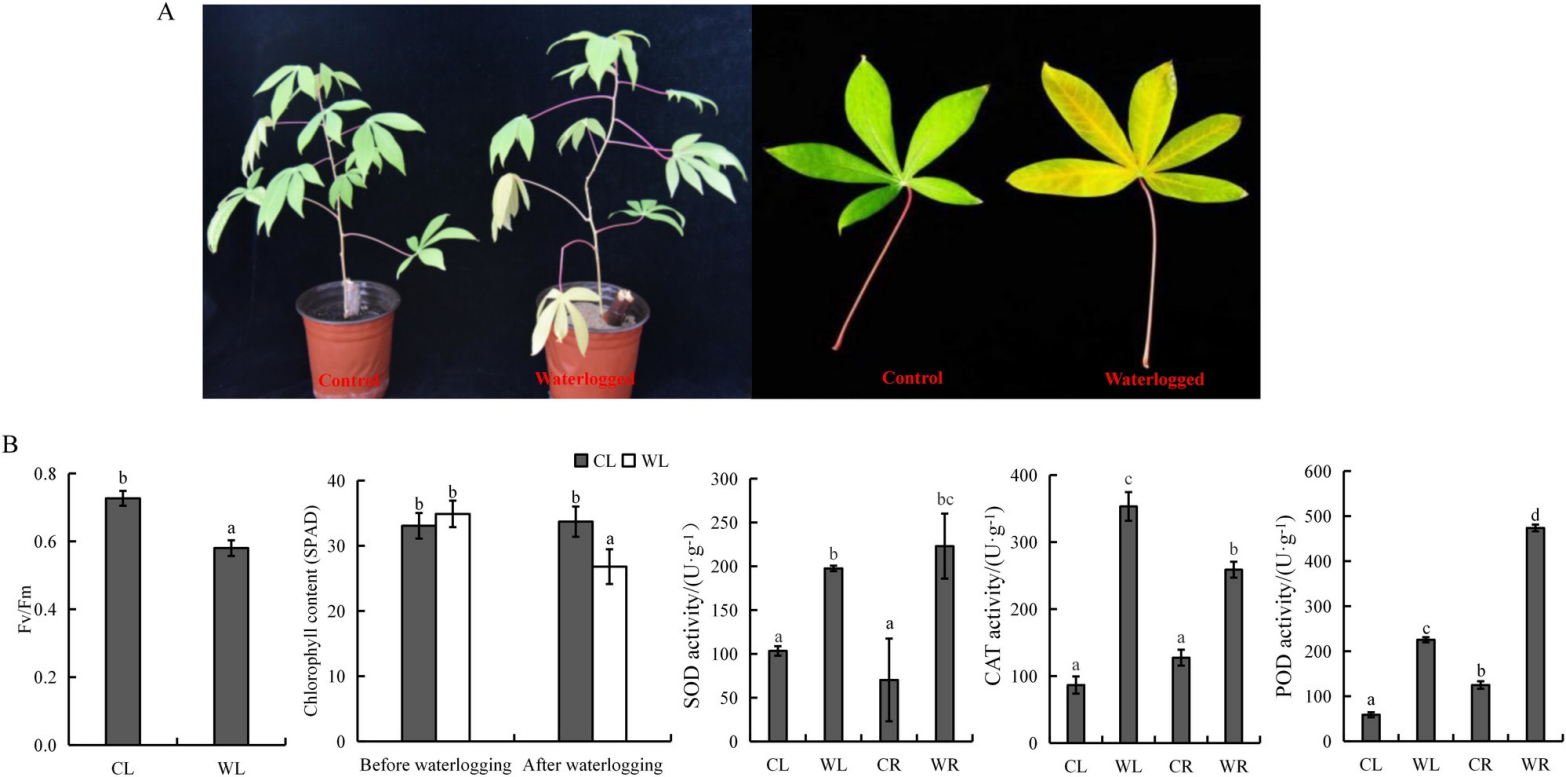
Morphological and physiological changes of cassava in response to waterlogging stress. (**A**) Changes of phenotypes and stem base leaves in control and waterlogged seedlings. (**B**) Determination of photosynthetic indexes and antioxidant enzymes in the leaves and roots under waterlogging stress. The photosynthetic indexes included maximum quantum yield (*Fv/Fm*) and chlorophyll content (SPAD). The antioxidant enzymes are superoxide dismutase (SOD), catalase (CAT) and peroxidase (POD). Different letters indicate significant differences from the control (*P* < 0.05). Bars indicate standard error (n = 3).

We compared the morphological changes in control and waterlogged plants after 6 days of waterlogging stress. No morphological changes were observed in the control plants, which had green leaves and upright stems. However, the leaves of the stem base of the waterlogged plants were withered and yellow, although the young leaves remained green (Fig 1A).

To investigate the photosynthetic ability of cassava plants under waterlogging stress, the chlorophyll content and *Fv/Fm* were determined. The chlorophyll content decreased by 20% under the waterlogging treatment (Fig 1B). The *Fv/Fm* values of the waterlogged samples also significantly decreased compared with those of the CL samples. In the WL samples, the *Fv/Fm* ratio was 0.5–0.6, while in the CL samples, the *Fv/Fm* ratio was approximately 0.7 or higher (Fig 1B). The observed decrease in *Fv/Fm* values may be associated with the sensitivity of the photosynthetic apparatus to waterlogging stress.

The activities of SOD, POD and CAT were measured for the CL, WL, and CR (roots under nonwaterlogged conditions) samples and the WR (roots under waterlogged conditions) samples. The activity of three antioxidant enzymes increased in the WL and WR samples. The SOD activity in the WL and WR samples reached values that were 1.9-fold and 3.2-fold higher than those in the CL and CR samples, respectively (Fig 1B). The activity of CAT in the WL samples reached 353 U·g^-1^, which was more than four times that in the CL samples (Fig 1B). The POD activity in the WR samples was 3.8 times higher than that in the CR samples (Fig 1B).

### 3.2 Waterlogging leads to extensive transcriptomic reprogramming

**Fig 2:**
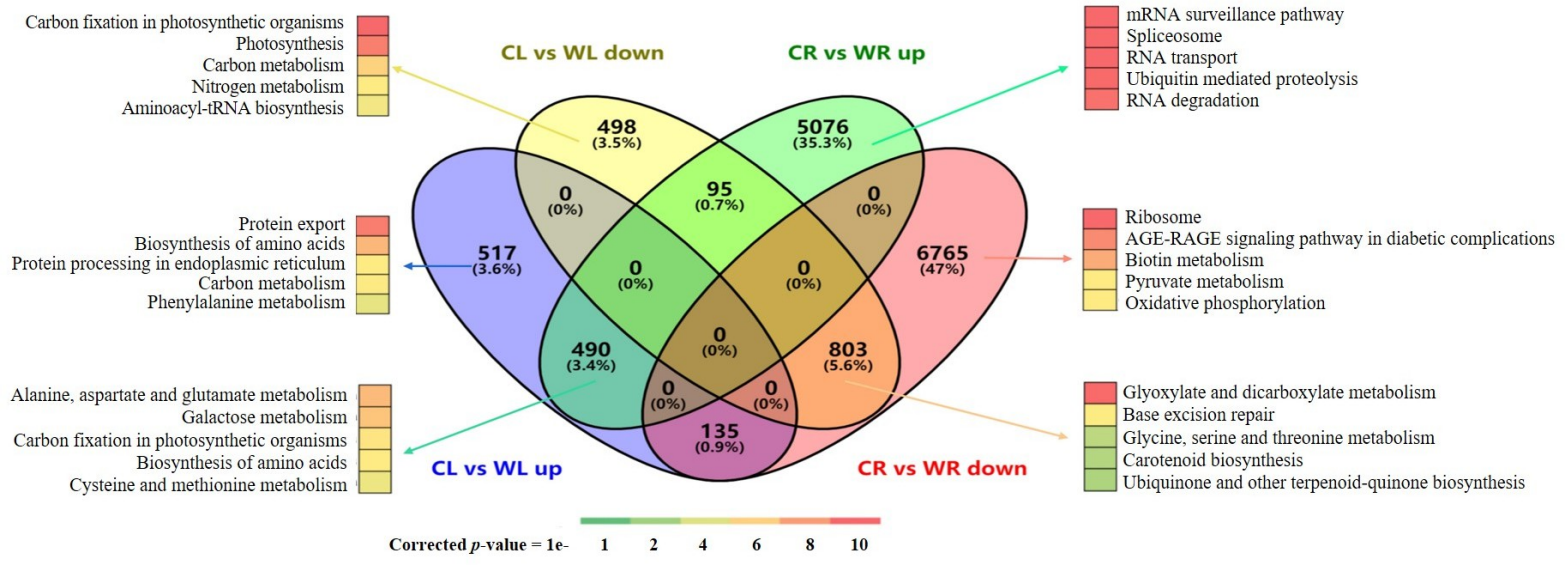
Venn diagrams of the differentially expressed genes under waterlogging treatment in the leaf and root samples (FDR corrected p-value of ≤ 0.01 and fold change ≥ 2) and their respective top five most significantly enriched KEGG pathways. The numbers of DEGs exclusively up- or down-regulated in one tissue are shown in each circle. The numbers of DEGs with a common or opposite tendency of expression changes between the two tissues are shown in the overlapping regions. Only the top 5 enriched pathway terms are shown.

After removing the unknown reads (those whose proportion of undetermined bases was > 10%), low-quality reads and reads that contained adapters and at least 39.4 million clean reads were obtained for each sample (Table 1). The clean reads were subsequently mapped to the reference genome, with the mapping ratio varying from 79.4% to 86.1%. More than 78% of the reads were uniquely mapped (Table 1). With a fold-change ≥ 2 and a FDR corrected *p*-value of < 0.01 used as screening criteria, 15902 genes were identified as differentially expressed in at least one tissue between the nonwaterlogged conditions and waterlogged conditions (Fig 2, S2 Table). Among them, 13364 differentially expressed genes (DEGs) were found in the CR vs WR pairwise comparison, and 2538 DEGs were found in the CL vs WL comparison. We discovered that there were many more DEGs in the CR vs WR comparison than in the CL vs WL comparison, suggesting that the waterlogging response is more complex in the roots than in the leaves. This is consistent with findings for *Taxodium* ‘Zhongshansa’ [31]. A total of 1523 genes were found to be shared between the two tissues, of which 625 showed opposite expression responses. Additionally, 11841 genes (5076 upregulated and 6765 downregulated) were exclusively differentially expressed between the CR and WR samples, and the expression levels of 1015 genes (517 upregulated and 498 downregulated) exclusively changed between the CL and WL samples (Fig 2, S2 Table).

**Table 1:** Total number of sequencing reads obtained from each sample.

Sample name	Total Reads	Mapped Reads	Uniq Mapped Reads	Multiple Map Reads
CL1	40,918,904	35,153,610(85.91%)	34,569,539(84.48%)	584,071(1.43%)
CL2	47,888,734	41,180,289(85.99%)	40,468,518(84.51%)	711,771(1.49%)
CL3	45,846,222	39,494,069(86.14%)	38,830,063(84.70%)	664,006(1.45%)
CR1	39,610,692	33,168,267(83.74%)	32,547,312(82.17%)	620,955(1.57%)
CR2	40,630,974	33,601,548(82.70%)	32,933,270(81.05%)	668,278(1.64%)
CR3	43,169,994	34,980,971(81.03%)	34,327,845(79.52%)	653,126(1.51%)
WL1	39,464,384	33,934,945(85.99%)	33,361,618(84.54%)	573,327(1.45%)
WL2	40,027,610	34,443,486(86.05%)	33,897,841(84.69%)	545,645(1.36%)
WL3	41,557,772	35,653,680(85.79%)	35,037,818(84.31%)	615,862(1.48%)
WR1	40,983,672	32,810,482(80.06%)	32,218,760(78.61%)	591,722(1.44%)
WR2	47,124,748	37,432,143(79.43%	36,741,146(77.97%	690,997(1.47%)
WR3	42,694,908	34,396,57(80.56%)	33,762,811(79.08%)	633,346(1.48%)

**Note:** CL: leaves under nonwaterlogged conditions; CR: roots under nonwaterlogged conditions; WL: leaves under waterlogged conditions; WR: roots under waterlogged conditions.

**Fig 3:**
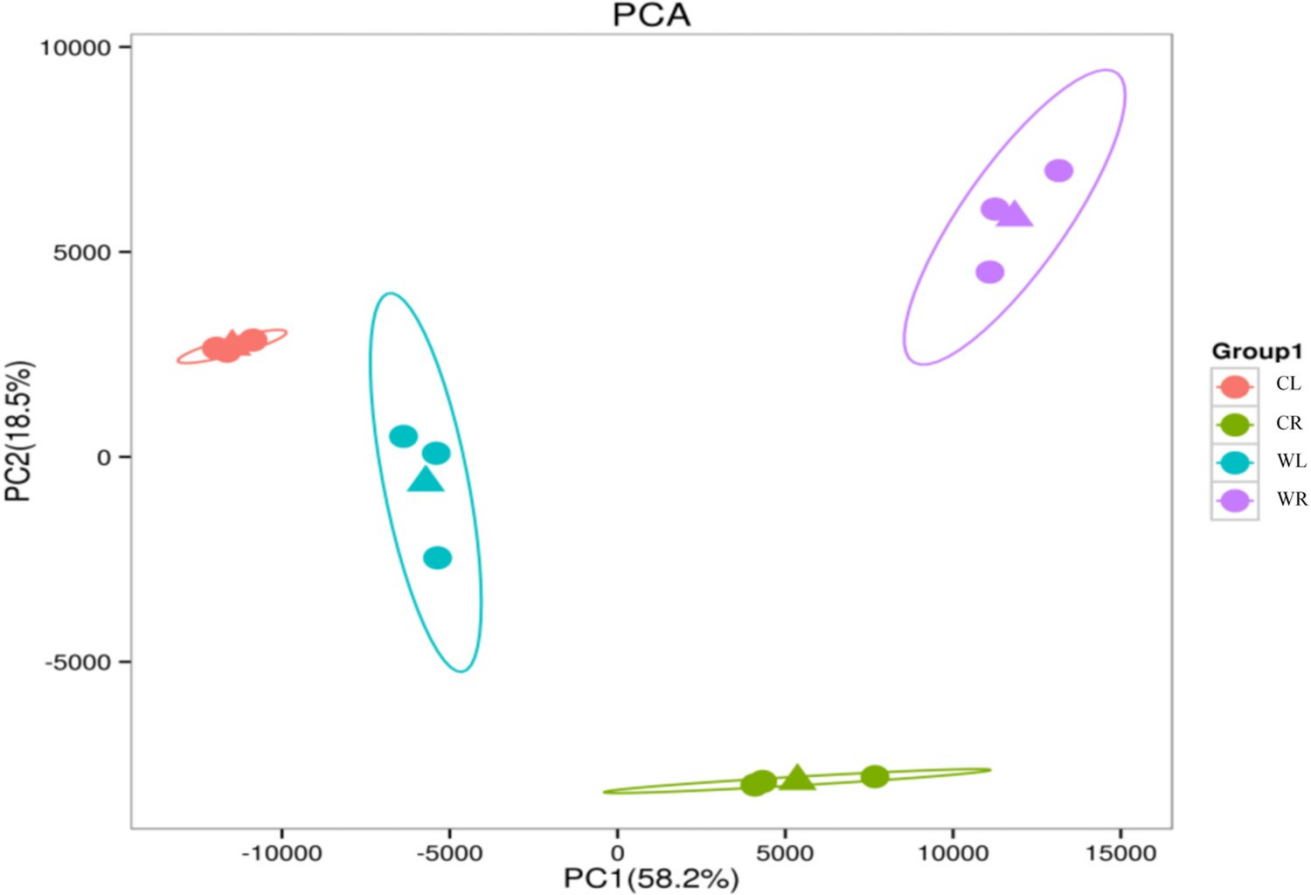
Principal component analysis of transcriptome data. Principal component analysis was applied to differentially expressed contigs identified in CL, CR, WL and WR. The triangles mean cluster centers.

Principal component analysis (PCA) was used to visualize the overall changes in gene expression in the different treatments. The first two principal components, which explained 76.7% of the total variance (58.2% by the first component, 18.5% by the second component), showed that the waterlogged tissues and nonwaterlogged tissues were dissimilar. Moreover, the replicates showed a high degree of similarity for all four treatments (Fig 3).

### 3.3 Functional annotation of waterlogging-responsive DEGs

To further characterize the expression changes discussed above, we compared the enriched KEGG pathways for DEGs between the two tissues, the results of which indicated some tissue-specific or highly performed functions (Fig 2). The following pathways were highly enriched in the DEGs whose expression was upregulated: ‘alanine, aspartate and glutamate metabolism’; ‘galactose metabolism’; and ‘carbon fixation in photosynthetic organisms’. However, other pathways were highly enriched in the DEGs whose expression was typically downregulated in both tissues, including ‘glyoxylate and dicarboxylate metabolism’; ‘base excision repair’; and ‘glycine, serine and threonine metabolism’. The pathways ‘protein export’, ‘biosynthesis of amino acids’, and ‘protein processing in endoplasmic reticulum’ were highly enriched in the DEGs whose expression was upregulated specifically in the CL and WL samples, while the ‘mRNA surveillance pathway’, ‘spliceosome’ and ‘RNA transport’ pathways were dramatically enriched in DEGs whose expression was specifically increased in the CR and WR samples. The DEGs involved in all aspects of photosynthesis, which were associated with ‘carbon fixation in photosynthetic organisms’, ‘photosynthesis’, ‘carbon metabolism’ and ‘nitrogen metabolism’, were listed as the top four enriched pathways in the CL and WL samples. The ‘ribosome’ pathway was the pathway most enriched by DEGs whose expression was downregulated specifically in the CR and WR samples.

### 3.4 Analysis of DEGs related to the photosynthesis pathway

**Fig 4:**
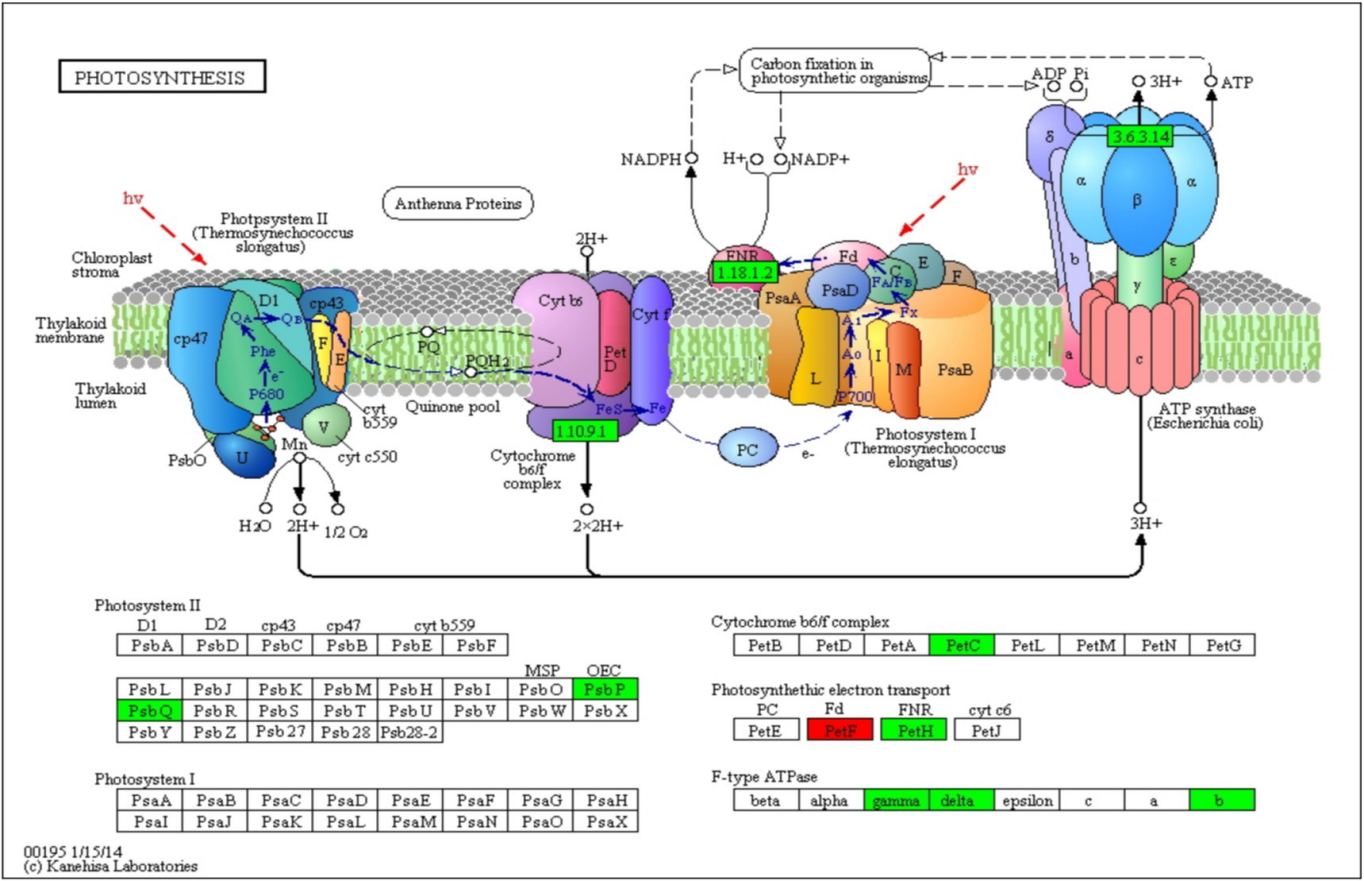
Differentially expressed genes mapped to the photosynthesis pathway in the leaves. The known pathways were obtained from the KEGG database. Red squares denote upregulated genes, and green squares denote downregulated genes.

PSI, PSII, the cytochrome b6/f complex, photosynthetic electron transport, and F-type ATPase are key components in the photosynthetic pathway. The comparison between the CL and WL libraries revealed 12 DEGs related to the photosynthesis pathway, including three genes related to photosystem II (PSII), four related to photosynthetic electron transport, one related to the cytochrome b6/f complex, and four related to the F-type ATPase. DEGs involved in PSII, the cytochrome b6/f complex and F-type ATPase were downregulated in the leaves when cassava was under waterlogging stress, two upregulated and two downregulated DEGs were related to photosynthetic electron transport (Fig 4, S3 Table). The malfunction of PSII reduced the efficiency of electron transport for photosynthetic reactions, which could result in substantive accumulation of reactive oxygen species (ROS) and further reduced PN under WL. Additionally, 12 genes involved in photosynthetic activities may be associated with the differences in leaf color and may cause a decrease in the *Fv/Fm* ratio.

### 3.5 Analysis of DEGs related to the glycolysis pathway

**Fig 5:**
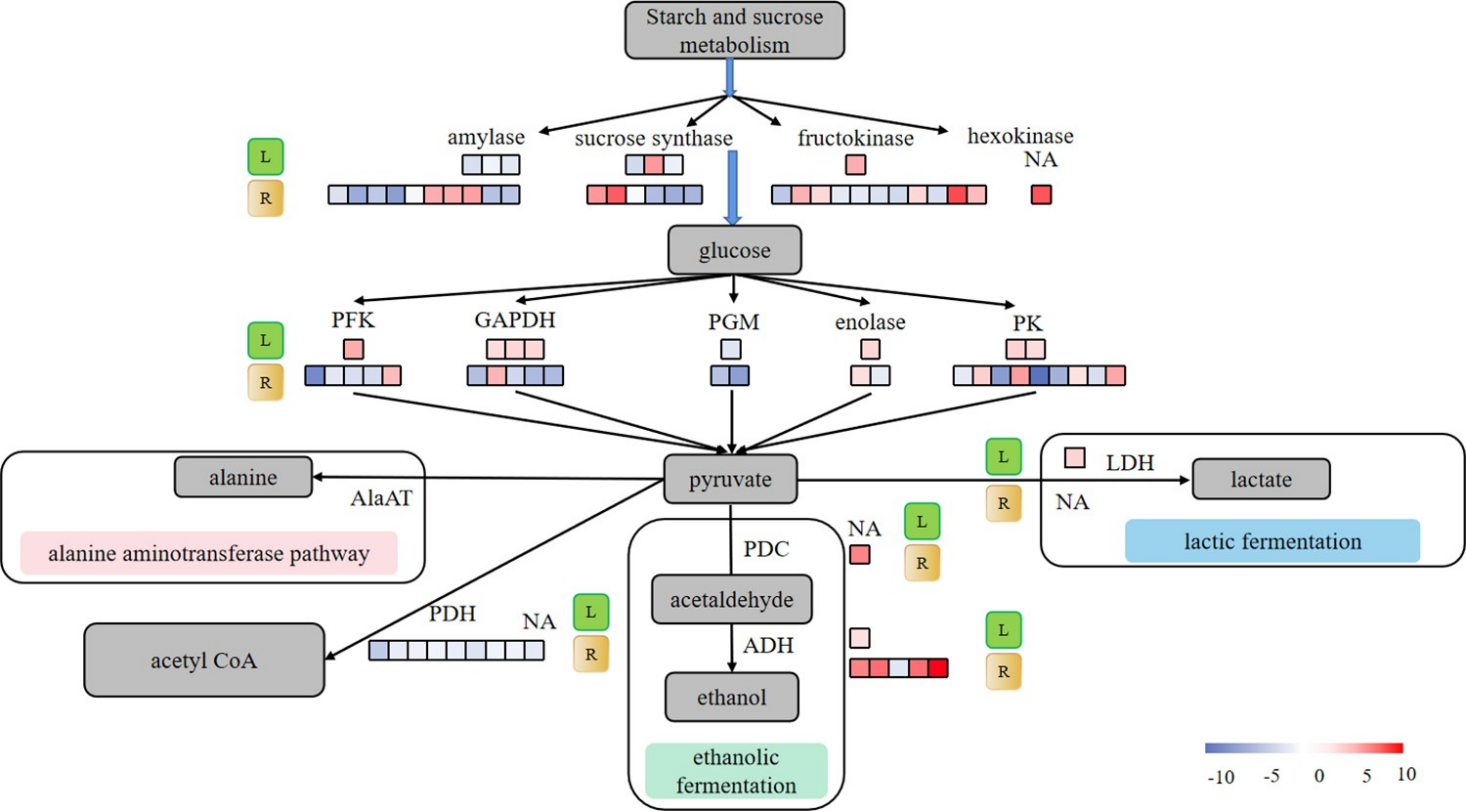
Schematic diagram of glycolysis and fermentation proposed during plant waterlogging stress. AlaAT, alanine aminotransferase; GAPDH, glyceraldehyde 3-phosphate dehydrogenase; PDC, pyruvate decarboxylase; ADH, alcohol dehydrogenase; PFK, 6-phosphofructokinase; PK, pyruvate kinase; LDH, lactic dehydrogenase; PDH, pyruvate dehydrogenase; PGM, phosphoglucomutase. L, leaves; R, roots; The color of the boxes represents the fold-change value of DEGs. The number of boxes represents the number of DEGs. NA indicates that there are no DEGs encoding the enzyme. The pathway was designed based on KEGG pathways (http://www.kegg.jp/kegg/pathway.html).

In both the roots and leaves of waterlogged cassava, many genes with potential roles in glycolysis and fermentation were identified as displaying a significant transcriptional response to waterlogging stress (Fig 5, S4 Table). A total of 113 DEGs (47 upregulated and 66 downregulated) were annotated as encoding enzymes involved in the glycolysis/gluconeogenesis pathway according to their KEGG classification (S4 Table). The expression of most of the DEGs was upregulated in the WL samples compared to the CL samples and were involved in enzymes such as glyceraldehyde 3-phosphate dehydrogenase (GAPDH), phosphofructokinase (PFK), enolase, alcohol dehydrogenase (ADH), L-lactate dehydrogenase (LDH) and pyruvate kinase (PK). However, in the WR samples, the expression of major glycolysis- and fermentation-related genes in response to waterlogging stress decreased, indicating a decrease in throughput of these pathways (Fig 5).

### 3.6 Analysis of DEGs related to the galactose metabolic pathway

**Fig 6:**
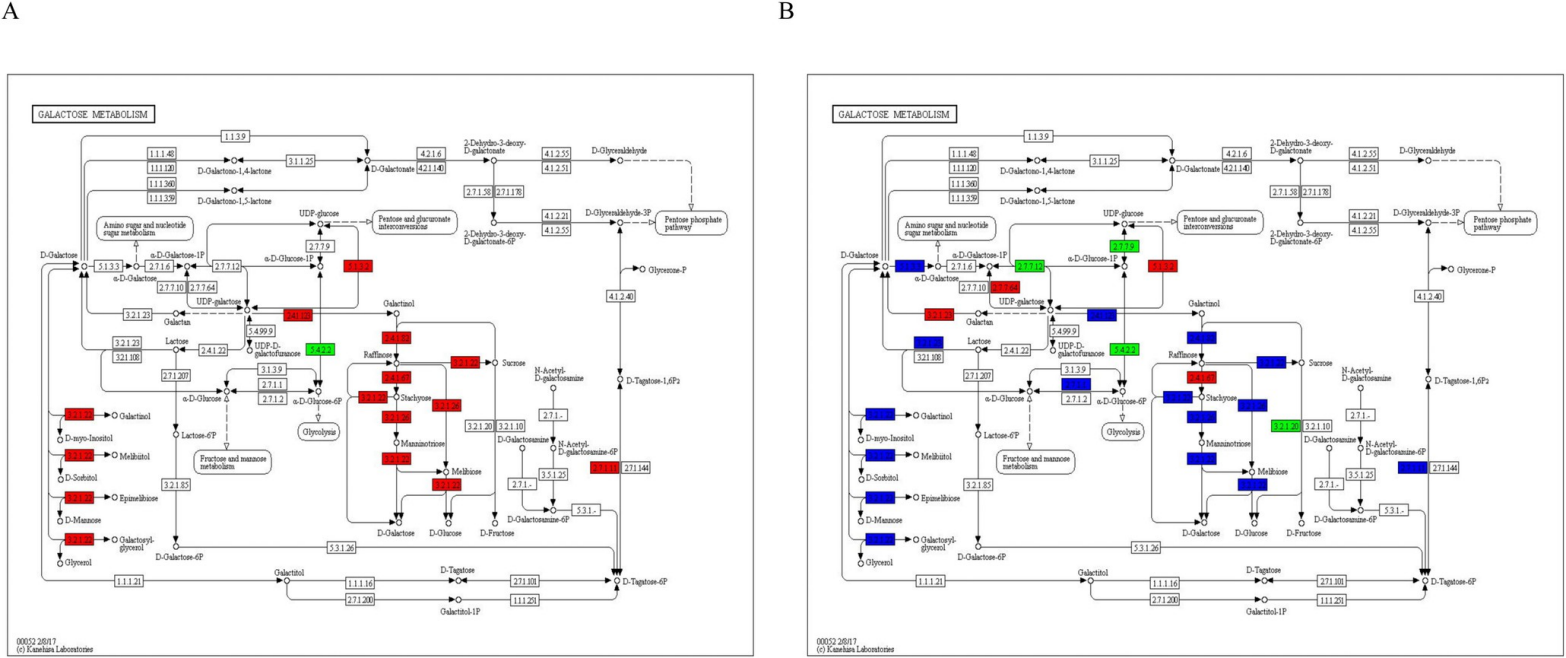
Analysis of DEGs involved in the galactose metabolic pathway in the leaves and roots. Pattern diagram of the lactose metabolic pathway in the (**A**) leaves and (**B**) roots. The red squares indicate upregulated genes, the green squares indicate downregulated genes, and the blue squares indicate both upregulated and downregulated genes. The pathway was designed based on KEGG pathways (http://www.kegg.jp/kegg/pathway.html).

A comprehensive analysis of the DEGs in the leaves and roots involved in the galactose metabolism pathway identified two and four genes in the leaves and roots that encode glucuronosyltransferase (inositol galactoside synthase) (Fig 6, S5 Table). This protein catalyzes the synthesis of inositol galactoside from UDP-galactose and myoinositol, which is the first key step in the synthesis of raffinose family oligosaccharides (RFOs) and the most critical regulatory step in RFO synthesis. The expression difference levels of two and three genes increased successively in leaves and roots, and one gene was downregulated in the roots (Fig 6, S5 Table). The analysis identified four and five genes encoding inositol galactoside-sucrose galactosyltransferase (raffinose synthase) in leaves and roots, respectively. This protein mainly catalyzes the synthesis of raffinose from inositol galactoside and sucrose. One and three genes encoding *β*-*D*-fructofuranoside were identified in the leaves and roots and mainly catalyze the decomposition of stachyose and raffinose into melibiose and the decomposition of sucrose into glucose and fructose. We also found two genes in the roots encoding *β*-galactosidase (*β*-GAL), which mainly catalyzes the decomposition of lactose and raffinose into melibiose (Fig 6B, S5 Table). Two of the main pectin deglycosylating enzymes that participate in this process are alpha galactosidases (*α*-GAL) (EC:3.2.1.22) and *β*-GAL [32]. In this study, four *α*-GAL DEGs were identified, two of which were upregulated after waterlogging in the roots, while two of them were downregulated. One *β*-GAL gene was identified and downregulated in the roots. Two *α*-GAL DEGs were upregulated in the leaves (Fig 6). One alpha glucosidase (*α*-Glu) gene, which is involved in cellulose degradation, was downregulated in the roots (S5 Table).

### 3.7 Responses of transcription factors (TFs) to waterlogging stress

**Fig 7:**
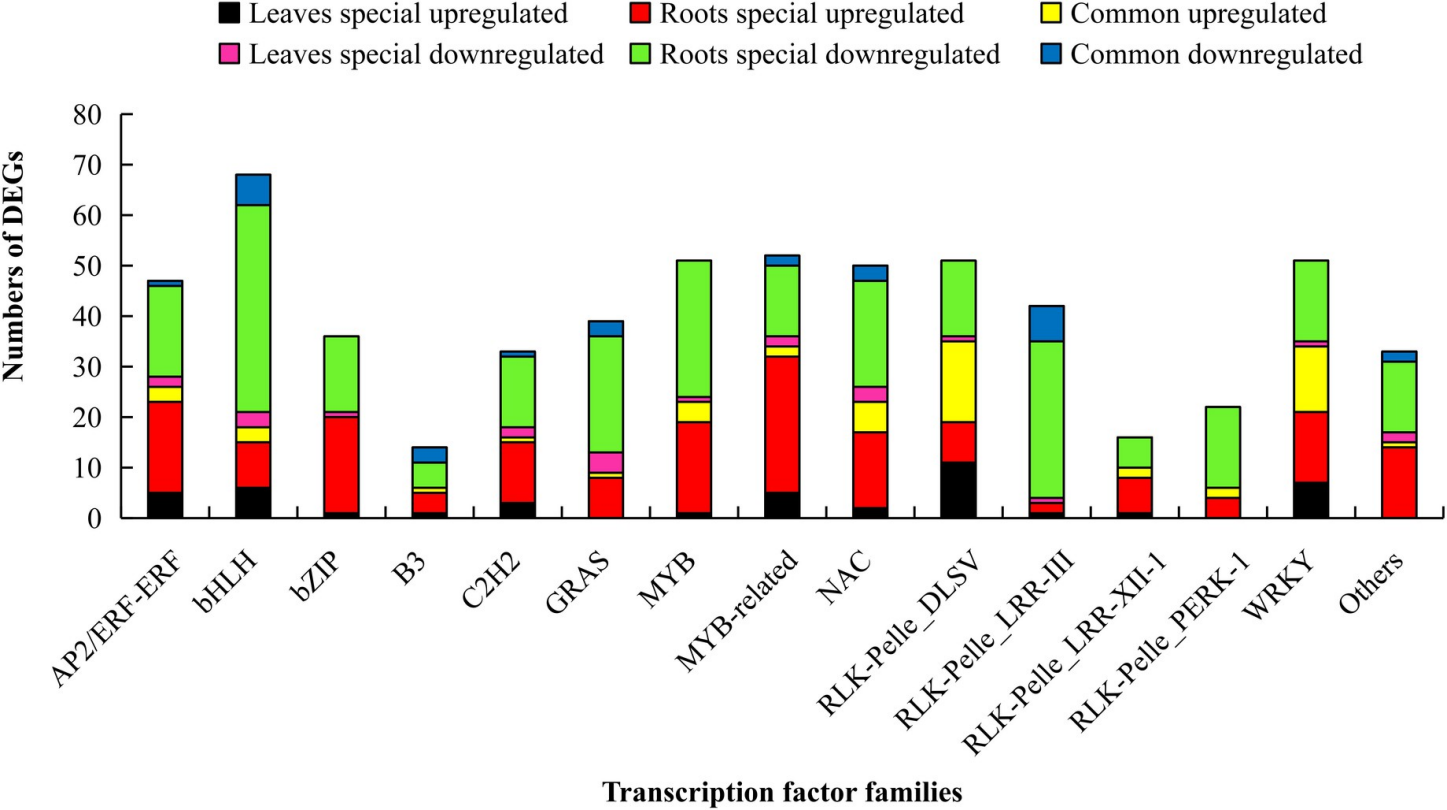
Response of transcription factors to waterlogging. Graphical representations of waterlogging-regulated transcription factors based on their assigned protein families. Different colors represent the different sources of DEGs, and the length of the bar represents the number of DEGs.

TFs are key regulators of target gene expression in response to various biotic or abiotic stresses by binding to specific cis-acting elements in these gene promoters [33]. In this study, a total of 605 waterlogging-regulated TFs were identified according to their assigned gene families (Fig 7, S6 Table). Of these, 84 TFs (55 upregulated and 29 downregulated) were commonly expressed in the leaves and roots, whereas 66 TFs (43 upregulated and 23 downregulated) were expressed in the leaves only, and 455 TFs (179 upregulated and 276 downregulated) were expressed only in the roots. These data strongly suggest that transcriptional regulation occurs in response to waterlogging. Remarkably, genes belonging to the MYB, WRKY, NAC, and AP2/ERF families encode most of the differentially expressed TFs, implying that these genes have important roles in waterlogging stress responses (Fig 7, S6 Table).

### 3.8 RNA-seq results validated by qRT-PCR

**Fig 8:**
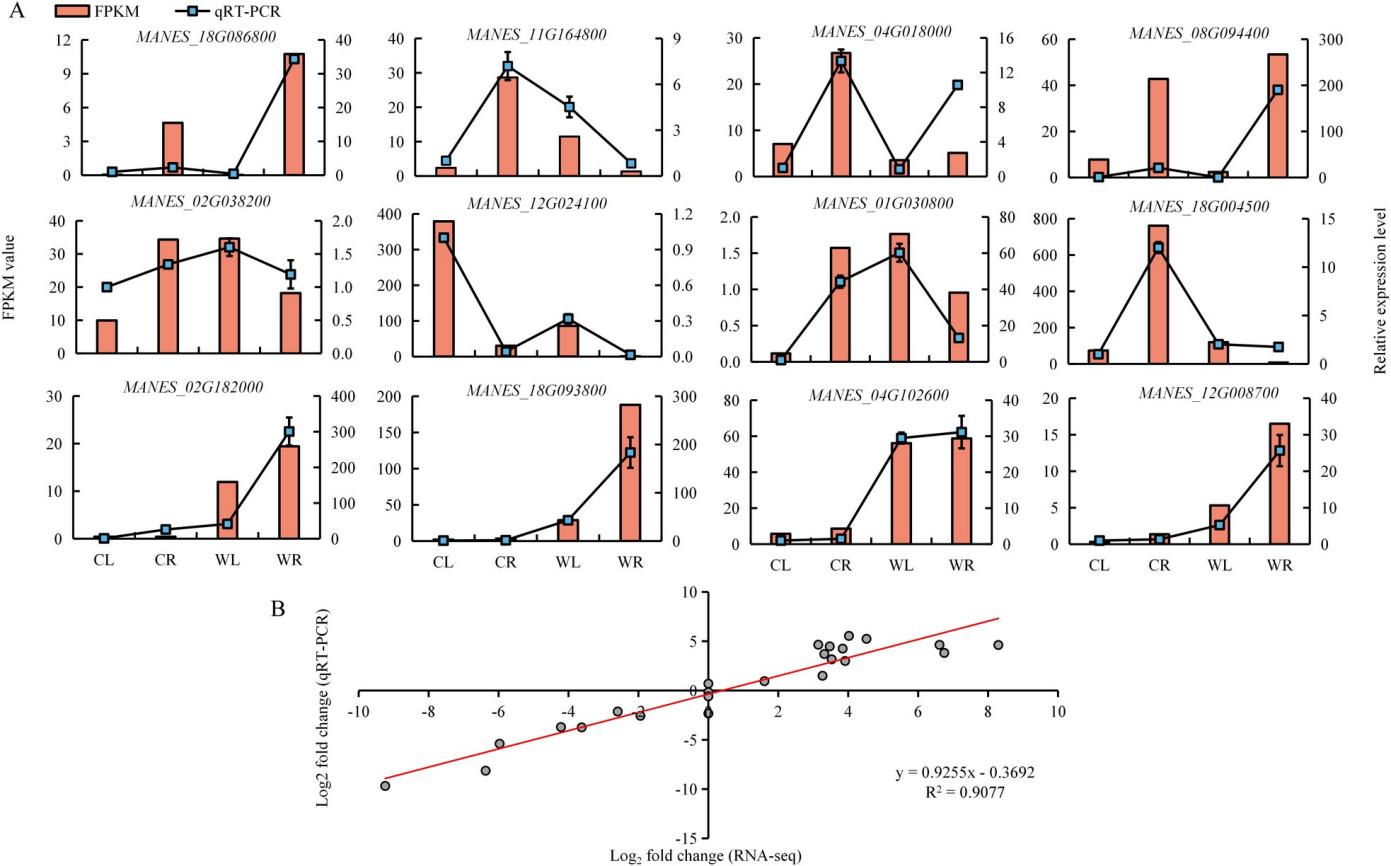
The expression patterns of 12 selected genes were verified by qRT-PCR, and the correlation between RNA-seq and qRT-PCR in the leaves and roots was verified. (**A**) The broken line represents the qRT-PCR results, and the bar graph represents the FPKM of RNA-seq. (**B**) The gene fold change log_2_ values (Y-axis) were plotted against the qRT-PCR fold change log_2_ values (X-axis). Each value denotes the mean relative level of expression of three biological replicates.

To verify the reliability of the RNA-seq data, we selected twelve genes for further investigation using qRT-PCR methods (Fig 8A, S1 Table). The results showed that the expression patterns determined via qRT-PCR were highly consistent with the RNA-seq data, with a relative R^2^ of 0.9077 (Fig 8B).

## 4. Discussion

Substantial progress has been made in understanding waterlogging or low-oxygen-stress response mechanisms at the transcriptional level in Arabidopsis [34, 35] and in various crop species, such as rice [36], rape [37], cucumber [38, 39] and maize [40], using transcriptomic approaches. These studies indicated that genes regulated by low oxygen availability are extremely diverse among species and are involved in ethylene synthesis, glycolysis, ethanol fermentation, carbohydrate catabolism, photosynthesis and galactose metabolism.

### 4.1 Comparisons of transcriptomes between leaves and roots

Waterlogging stress is different from complete submergence in that only the lower and subsoil portions of affected plants are subjected directly to the stress. In this study, comparison of a transcriptional response to waterlogging stress between the aboveground tissues (leaves) and belowground tissues (roots) revealed a high number of DEGs during the same period. These data can be used to discern among the enriched gene function categories, which can be reasonably explained by the functional differences between the two tissues. A previous report showed that the ability to tolerate hypoxic stress in the leaves and roots could be genetically based [31, 41], and the anaerobic induction of most known anaerobic proteins (ANPs) was root specific.

Genes associated with functional categories related to mRNA surveillance, RNA transport and degradation were most highly enriched in the roots, but they were also dramatically enhanced in the WR samples, which indicated a large scale of energy savings under hypoxic conditions. However, few studies have analyzed the role of these genes in the waterlogging response. In addition, the genes that were involved with ribosomes and whose expression was downregulated were the most enriched in the WR samples, suggesting that waterlogging decreased ribosome biosynthesis. Ribosomes are large and complex molecular machines found within all living cells in plants and serve as the primary sites of biological protein synthesis or translation [42]. Downregulation of ribosomal genes has been found in several plant species exposed to heavy metal stress [43, 44]. Our results suggest that there is a common ribosome biosynthesis network between waterlogging and heavy metal stress in cassava.

Moreover, the disruption in aerobic respiration caused by waterlogging may inhibit the tricarboxylic acid (TCA) cycle and activate glycolysis and fermentation pathways at the whole-plant level, resulting in the accumulation of amino acids closely derived from glycolysis intermediates (glycine, serine, threonine, etc.) and a decrease in TCA cycle intermediate-derived amino acids (asparagine, aspartic acid, glutamine, glutamic acid, etc.) [45]. In this study, as expected, the genes related to ‘alanine, aspartate and glutamate metabolism’ and ‘glycine, serine and threonine metabolism’ were highly enriched in both tissues, suggesting that pathways related to carbohydrate and amino metabolism were activated when cassava was exposed to hypoxic conditions. This finding was consistent with those in alfalfa [46].

### 4.2 Effects of waterlogging stress on photosynthesis

Chlorosis is a typical symptom that inevitably occurs after severe waterlogging. Because waterlogging causes water saturation and subsequent rapid closure of stomata, a high concentration of O_2_ cannot be released out, and photosynthetic electron transportation is blocked in chloroplasts [47, 48].

Waterlogging at the plant stage of cassava plants caused significant decreases in chlorophyll content and the *Fv/Fm* ratio and caused premature senescence of leaves (Fig 1). These results were consistent with those of previous studies in which waterlogging-induced chlorophyll loss was associated with a decrease in photosynthetic activity [49]. Interestingly, the *Fv/Fm* ratio was inconsistent with the findings of Guide and Soldatini [50], who observed that after 6 days of waterlogging, the *Fv/Fm* ratio remained unchanged and photosynthesis continued to decrease in the absence of stomatal closure in soybean. DEGs relevant to photosynthesis were abundant in the leaves compared with the roots. In this study, the top two enriched pathways by genes whose differential expression was specifically downregulated in the WL samples were associated with photosynthesis pathways, indicating a possible reduction in the photosynthesis rate (Fig 4, S3 Table). These DEGs were involved in PSII and the photosynthetic electron transport chain (cytochrome b6-f complex, ferredoxin, transporting ATPase subunit, etc.). The reaction centers of PSII in chloroplast thylakoids are also the major generation sites of ROS and are largely affected by abiotic factors such as low oxygen concentrations [37, 51]. In addition, the expression levels of genes encoding rubisco and rubisco activase, which are involved in carbon-fixing reactions, were also downregulated, suggesting that waterlogging stress not only inhibits photosynthetic reactions but also reduces the efficiency of CO_2_ fixation in cassava. Similar waterlogging responses in other crop species have been previously reported [37, 52].

### 4.3 Effects of waterlogging stress on glycolysis pathway

Generally, plants receive their essential energy supply through glycolysis and ethanol fermentation when facing energy shortages caused by waterlogging stress [53]. In this study, many genes, including well-known hypoxia-related genes associated with glycolysis and fermentative processes, were identified as having a significant transcriptional response to waterlogging stress in both tissues, which indicated that the pathway was activated to maintain ATP production under hypoxic conditions. Most ANPs have been identified as enzymes involved in the glycolysis or sugar phosphate pathways that are needed to maintain energy production under waterlogging conditions [54, 55].

Fermentation is a process of energy conversion necessary during waterlogging stress, during which the expression of some anaerobic genes, such as *PDC* and *ADH*, is upregulated [56, 57]. Overexpression of *PDC1* and *PDC2* in Arabidopsis improves survival under low-oxygen conditions [58]. A similar result was obtained by Rivoal et al. in rice [59]. ADH activity has been reported to increase under anoxia, and compared with wild-type plants, maize mutants deficient in ADH activity are more sensitive to waterlogging stress [60]. In this study, the expression of four out of five *ADH* genes was significantly upregulated in the roots under waterlogging stress (S4 Table), and the expression of one *PDC* gene was also significantly upregulated. These results suggest that energy was produced under hypoxic conditions by the activation of alcoholic fermentation. The expression of none of the genes encoding PDC changed in the CL vs WL comparison. This finding was consistent with findings for *Taxodium* ‘Zhongshansa’ [31] and gray poplar [45]. In addition to PDC and ADH, LDH is also involved in the response to low-oxygen conditions and is activated in the initial stages of root hypoxia in many plant species. In a study on gray poplar, LDH transcripts were also rather abundant during the initial reaction to oxygen deprivation but decreased after approximately 5 h due to the decrease in cytosolic pH caused by lactic acid [45]. In this study, the expression of an *LDH* gene was significantly upregulated in the WL samples, while in the WR samples, it was unchanged. Additional quantitative real-time PCR experiments are needed to confirm the importance of *LDH* in the roots of cassava during waterlogging tolerance.

### 4.4 Effects of waterlogging stress on galactose metabolism

The energy metabolism pathway is not only related to the alcohol dehydrogenase-catalyzed step of glycolysis but also associated with starch and sucrose metabolism and the galactose metabolism pathway. Accumulating data suggest that low oxygen concentrations play a key role in the induction of hypoxia metabolism, such as the expression of genes triggering anaerobic fermentation, sugar utilization and antioxidant defense [61, 62]. We found that after waterlogging treatment, the expression levels of major genes related to galactose metabolism in the leaves and roots were upregulated. Raffinose and galactitol, as ROS scavengers, can reduce oxidative damage under abiotic stress conditions [63]. Additionally, raffinose can be transported to chloroplasts to protect thylakoids and stabilize PSⅡ [64]. Both galactinol and raffinose accumulate at higher levels in plants in response to abiotic stresses [65]. They play a novel role in the protection of cellular metabolism from oxidative damage caused by salinity, chilling, or drought [66]. Under the condition of long-term waterlogging, the cell wall of plants will disintegrate before the whole cell disintegrates [67, 68]. Degradation of cell wall polysaccharides is a consequence of synergistic action among several key cell wall modifying enzymes, including polygalacturonase (PG), pectin methyl esterase (PME), *β*-GAL and cellulase (CEL) [69]. In our study, two *β-GAL* genes in the roots and one *β-GAL* gene in the leaves were downregulated, suggesting that the cell wall degrades slowly under waterlogging stress (S5 Table).

### 4.5 Changes in the expression of TFs

Previous studies have reported that MYB TFs are closely related to the primary and secondary products of plant morphogenesis and to the metabolic regulation of plant resistance [55, 70–72]. The members of this TF family are also known to trigger *ADH* gene. For instance, the induction of *AtADH1* expression is tightly coupled to the initial increase in *AtMYB2* transcripts [73]. Research on wheat has shown that the expression of *TaMyb1* is strongly induced in the roots under hypoxic conditions [74]. We found 23 MYB- and 34 MYB-related TFs in the upregulated group, indicating that these genes have potential roles in waterlogging stress responses and tolerance. ERF TFs belong to a plant-specific TF superfamily related to the stress response. Previous studies have shown that AP2/ERF TFs such as *Sub1A*, *HRE1*, *HRE2*, *Snorkel1*, *Snorkel2*, and *RAP2*.*2* are important for tolerance to plant hypoxia caused by waterlogging [75–77]. For instance, overexpression of *SK1* and *SK2* significantly enhances the waterlogging tolerance of rice and is characterized by excessive elongation of internodes [75]. Hinz reported that constitutive overexpression of *HRE1* or *HRE2* could rapidly induce the expression of hypoxia-responsive genes, such as *PDC* and *ADH*, to enhance waterlogging tolerance in Arabidopsis [76]. Under hypoxia, the presence of *RAP2*.*12* in the nucleus may lead to not only relocalization of the existing protein but also de novo synthesis [78]. In this study, the expression of 26 and 21 *AP2/ERF* genes was upregulated and downregulated, respectively, under waterlogging stress. Whether the TF changes are related to cassava waterlogging tolerance requires further demonstration. Additionally, members of the WRKY and NAC TF families may also be associated with the transcriptional regulation of genes involved in the waterlogging response of cassava, as these TFs were overrepresented in the list of DEGs. These results were similar to those for soybean [79] and kiwifruit [11].

## 5. Conclusion

In this study, we used high-throughput sequencing to characterize the transcriptome responses in the roots and leaves of partially submerged cassava plants. In total, 15902 transcripts were identified as being differentially expressed. KEGG enrichment analysis provided comparisons of iCL vs WL and of CR vs WR. The results suggested that waterlogging stress mainly represses photosynthesis reactions in the leaves and improves energy savings in the roots. Amino acid metabolism also greatly changed in both tissues, and a nitrate-producing pathway may be induced to help maintain ATP levels. Furthermore, complex interactions between energy production and the antioxidant enzyme system were observed, suggesting that they have important roles in the waterlogging response. The changes in the expression of these genes involved in the waterlogging response might be regulated by the synthesis and perception of some TFs, such as ERFs, MYBs, WRKYs and NACs.

## Supporting information

S1 Table Gene description and primers used for qRT-PCR analysis. (XLSX)

S2 Table List of DEGs in the leaves and roots and the different sources of DEGs. (XLSX)

S3 Table DEGs information involved in photosynthesis pathway in the leaves under waterlogging stress. (XLSX)

S4 Table List of DGEs encoding enzymes involved in the glycolysis pathway in the leaves and roots. (XLSX)

S5 Table List of DGEs encoding enzymes involved in the galactose pathway in the leaves and roots. (XLSX)

S6 Table List of waterlogging-regulated TFs in the leaves and roots. (XLSX)
